# Biomarkers for Non-Small Cell Lung Cancer: From the Bench to the Bedside

**DOI:** 10.3390/jcm9103376

**Published:** 2020-10-21

**Authors:** Ramon Andrade de Mello, Giovanna Araújo Amaral

**Affiliations:** 1 Algarve Biomedical Centre, Department of Biomedical Sciences and Medicine University of Algarve (DCBM UALG), 8005-139 Faro, Portugal; 2 Division of Medical Oncology, Escola Paulista de Medicina, Federal University of São Paulo (UNIFESP), São Paulo 04037-004, Brazil; gioaraujoamaral@gmail.com; 3 Precision Oncology and Health Economics Group (ONCOPRECH), Post-Graduation Program in Medicine, Nine of July University (UNINOVE), São Paulo 01525-000, Brazil

Lung cancer (LC) is inarguably one of the biggest battles to be fought in the field of oncology, and non-small cell lung cancer accounts for over 85% of all lung cancer cases. According to the World Health Organization’s Global Cancer Observatory, lung cancer is currently the third most common malignancy in the world and, in spite of a decreasing mortality since the 1990s, it remains among the most aggressive cancers to this day. Its global mortality in 2018 consisted of 18.6 deaths per 100,000 individuals, which accounts for 18.4% of all cancer deaths [1]. The trends in lung cancer mortality and incidence vary, however, they are in accordance with the stages of the smoking epidemic currently playing out in each country. In many underdeveloped countries in South America and Asia, where smoking habits have not yet been curbed by populational health strategies, mortality and incidence rates of lung cancer are increasing. Since underdeveloped countries account for a high percentage of the worldwide population, the global burden of lung cancer is set to increase in years to come, especially considering the lack of high technology treatment options in low- and middle-income countries. Another development in lung cancer epidemiology is the advent of women as a key population in this disease, due mostly to the increase in smoking in the female gender. Lung cancer mortality rates in American women increased by 600% from the 1930s to the early 21st century, and are currently decreasing at a slower pace when compared to the male statistics [2].

The toll of lung cancer on the patients is yet another cause for concern. Studies show that fatigue, loss of appetite and pain are present in more than 90% of lung cancer patients. This malignancy is correlated with a higher symptom burden and higher disease-related distress when compared to other cancers, significantly lowering the patients’ personal assessment of quality of life [3,4]. The economic burden of LC is also significant. A recent retrospective cohort study from South Korea shows that 5-year medical costs for lung cancer patients averages 36,000 dollars for patients who undergo surgery combined with chemo and radiotherapy [5]. A similar study conducted in the United States in 2005 showed an average of 2-year medical costs of 45,000 dollars for lung cancer patients [6]. Populational costs of lung cancer care amounted to USD 12.1 billion in the United States alone in 2010 [7].

In the 1960s, patients with lung cancer received surgery or radiotherapy, and patients with advanced lung cancer were only given supportive care. In the 1980s, chemotherapy was introduced for LC patients in clinical settings with cisplatin, which was later combined with other cytotoxic agents such as paclitaxel, docetaxel and gemcitabine. Cisplatin was replaced over the years with carboplatin, a drug with less toxic effects on the kidneys and central nervous system [8]. The main advancements in lung cancer treatment consisted of the development of target-therapies to accompany chemotherapy. Target-therapies became possible due to the advancement of research on the genetic and molecular landscape of the tumors, which enabled the identification of biomarkers, mutations most commonly found in lung cancer cells that serve as targets for treatment. The drug bevacizumab, an anti-angiogenic factor which targets VEGFR (vascular endothelial growth factor receptors), was approved by the Food and Drug Administration (FDA) in 2006 for LC treatment, inaugurating target-therapies for this malignancy. In 2013, the FDA approved another target-therapy drug, erlotinib, which targets EGFR (endothelial growth factor receptor). The targeting of EGFR advanced with the advent of second and third-generation inhibitors. Amongst the third-generation, osimertinib showed great treatment promise, and was approved in 2015. Inhibitors of the ALK (anaplastic lymphoma kinase) gene came into the scene in 2011 with the drug crizotinib. Newer ALK inhibitors include ceritinib, alectinib and brigatinib, which were approved in 2014, 2015 and 2017, respectively. Inhibitors of PD-L (programmed death ligand) started being used in 2015, with the monoclonal antibody nivolumab. Pembrolizumab and atezolizumab are newer examples of PD-L targeting antibodies, approved in 2016 [9]. In spite of the development of all of the aforementioned drugs for lung cancer treatment, 5-year survival rates for patients diagnosed with this malignancy are still low and the mortality rates remain high, as many challenges are yet to be overcome. Some of these challenges include the acquired resistance of the tumor cells to specific target-therapies and the necessity for developing sensible and specific molecular biomarkers to diagnose LC patients earlier and accurately predict prognosis [10].

The aforementioned scenario points to a great necessity of deepening the research on the subject of lung cancer, in order to increase medical knowledge of the genesis and progression of this tumor. The dissemination of research on lung cancer can translate into better clinical management of patients and development of new treatment technologies, decreasing the global burden of this malignancy. To this effect, the current issue of this journal addresses relevant topics regarding non-small cell lung cancer, such as molecular characterization of lung cancer cells for the identification of new biomarkers, review of the target-therapies that show the most promise in increasing patient survival, amongst other valuable discussions.
